# Voice in different phases of menstrual cycle among naturally cycling women and users of hormonal contraceptives

**DOI:** 10.1371/journal.pone.0183462

**Published:** 2017-08-22

**Authors:** Irena Pavela Banai

**Affiliations:** Department of Psychology, University of Zadar, Zadar, Croatia; Jagiellonian University, Poland, UNITED STATES

## Abstract

Previous studies have shown changes in women's behavior and physical appearance between the non-fertile and fertile phases of the menstrual cycle. It is assumed that these changes are regulated by fluctuations in sex hormone levels across the cycle. Receptors for sex hormones have been found on the vocal folds, suggesting a link between hormone levels and vocal fold function, which might cause changes in voice production. However, attempts to identify changes in voice production across the menstrual cycle have produced mixed results. Therefore, the purpose of this study was to investigate changes in sexually dimorphic vocal characteristics and quality of women’s voices in different phases of the cycle and to compare these with users of monophasic hormonal contraception. Voice samples (vowel phonation) of 44 naturally cycling women were obtained in the menstrual, late follicular (confirmed by LH surge) and luteal phases, and in 20 hormonal contraceptive users across equivalent stages of the monthly cycle. Results showed that voices of naturally cycling women had higher minimum pitch in the late follicular phase compared with the other phases. In addition, voice intensity was at its lowest in the luteal phase. In contrast, there were no voice changes across the cycle in hormonal contraceptive users. Comparison between the two groups of women revealed that the naturally cycling group had higher minimum pitch in the fertile phase and higher harmonics to noise ratio in the menstrual phase. In general, present results support the assumption that sex hormones might have an effect on voice function. These results, coupled with mixed findings in previous studies, suggest that vocal changes in relation to hormonal fluctuation are subtle, at least during vowel production. Future studies should explore voice changes in a defined social context and with more free-flowing speech.

## Introduction

Over the last two decades, there has been growing interest in various changes in women’s appearance and behavior across the menstrual cycle, especially in how the fertile period compares to other stages of the cycle. For example, previous studies have shown that during their fertile period, women have an increased preference for male masculine traits [1], extra-pair sexual interests and fantasies [2, 3], tendency to flirt with an attractive man [4], to wear attractive clothes that are more revealing, and to dress more provocatively [5, 6]. Women also take care more about their looks [6], consume fewer calories, and spend more money on beauty products when they are fertile [7]. Furthermore, Miller, Tybur & Jordan [8] reported that exotic dancers earn more money during their fertile days. This implies that men can detect subtle changes in the fertile period, and it is possible that they rely on olfactory and visual information. In line with this, previous studies showed that women's body odour changes across the cycle, being most attractive in the fertile period [9–12]. With regard to visual ovulation cues, Roberts et al. [13] reported that men chose women's photographs taken in the fertile phase as more attractive than photographs of the same women taken in the luteal phase. Similarly, Bobst and Lobmaier [14] showed that prototype faces of women in the fertile phase are more attractive.

Considering ultimate causes of these changes, it is assumed that they represent an adaptation with the purpose of finding an adequate partner and enhancing the genetic quality of any potentially resulting offspring [15] (but see [16]). With respect to proximate causes, it is assumed that these changes are caused by fluctuations in sex hormone levels across the cycle. Estrogen levels peak in the late follicular phase, which corresponds to the period of highest conception probability, while progesterone levels peak in the luteal phase. On the contrary, the menstrual phase is characterized by low levels of both sex hormones.

Receptors for sex hormones have been found on the vocal folds [17, 18], which suggests a link between hormone levels and vocal fold function. Hence, besides investigating olfactory and visual cues of ovulation, researchers have recently focused on auditory information by exploring voice changes across the cycle and the possibility that voice might similarly convey information about women’s fertility.

Generally, voice can convey valuable information about various characteristics of the speaker, such as sex, body shape and size [19], and emotional state [20]. It was also recently found that voice could provide information about genetic quality. It was found that voice attractiveness was related to fluctuating asymmetry, which is thought to reflect genotype's resilience to environmental stressors [21]. In contexts relevant to potential vocal changes across the menstrual cycle, increased estrogen levels during the late follicular phase result in thickening of the laryngeal mucosa and increased mucus production, while increased progesterone levels during the luteal phase result in drying of the laryngeal mucosa [22]. These proposed physiological changes in the laryngeal mucosa in relation to hormone fluctuation might cause changes in voice production, as well as in voice quality and perceived attractiveness. Indeed, research has revealed that attractiveness ratings of women's voices varied across the menstrual cycle, with higher ratings being found for voices recorded in the fertile phase [23–27], and lowest ratings for voices recorded in the menstrual phase [28]. This implies that voice is also a potentially valuable source of biologically important information such as women's fertility.

Considering which acoustic characteristics might reveal fertility, most researchers have focused on fundamental frequency of phonation (F_0_), which is perceived as voice pitch. F_0_ is based on the rate of vocal fold vibrations, which depends on the size, thickness and length of the vocal cords [22]. Women have smaller, thinner and shorter vocal cords than men, so their vocal cords vibrate at higher frequencies [22, 29]. Consequently, women have higher-pitched voices than men, which makes this acoustic characteristic a sexually dimorphic trait. Previous studies showed that F_0_ is related to ratings of attractiveness. For example, women with higher-pitched voices are perceived as more attractive [30–33], younger [34], more feminine [35], and more desirable spouses [36]. In addition, faces of women with higher-pitched voices are perceived as more attractive [37]. Furthermore, when presented with higher-pitched female voices, men visualize younger women with attractive and feminine faces [38].

The literature reviewed above suggests that voice pitch could change across the menstrual cycle, causing an increase in voice attractiveness during mid-cycle. However, attempts to detect proposed acoustic changes have produced mixed results. While Bryant & Haselton [23] found an increase in voice pitch in the fertile period, many other studies report no significant changes across the cycle [39–45]. Furthermore, in a recent study conducted by Tatar et al. [46] on a sample of Turkish women, voice pitch was higher in the follicular than in luteal phase, but did not differ significantly from the pitch recorded in the menstrual phase. To complicate matters further, Amir, Kichon-Rabin, & Muchnik [47], Fischer et al. [24], and Karthikeyan & Locke [25] found a decrease in voice pitch during the fertile phase; in the latter study, voice was rated as more attractive in the fertile period but these ratings did not correlate with cycle differences in pitch. This implies that other acoustic characteristics, which also change across the cycle, may be involved in how others perceive them. Indeed, researchers investigated changes of other sexually dimorphic acoustic characteristics, like pitch variability (F_0_ SD), which is an indicator of vocal stability. Higher values of F_0_ SD are found in female voice, which makes it less monotone and stable in comparison to male voice [29, 48, 49]. Previous study also revealed changes in F_0_ SD in response to attractiveness of potential partner, and competition against an attractive rival [50]. However, no changes in F_0_ SD were found across the cycle [23, 24].

Besides focusing on sexually dimorphic acoustic characteristics, parameters that signal vocal quality were also assessed in previous studies. These parameters include frequency perturbation (jitter), amplitude perturbation (shimmer) and harmonics to noise ratio (HNR). Higher values of jitter or shimmer, and lower HNR values, all indicate irregularity in vocal cord vibration, which is usually perceived as breathiness and vocal hoarseness. On the contrary, lower jitter and shimmer, and higher HNR values, are related to greater vocal quality. Chae et al. [41] and Tatar et al. [46] found higher jitter during the premenstrual period compared to the follicular phase, which suggests greater quality of voice in the fertile phase. Fischer et al. [24] reported lower HNR in the menstrual compared to the late follicular phase. On the contrary, Tatar et al. [46] found no difference between the menstrual and follicular phases, but higher HNR in the follicular phase compared to the luteal phase. Nonetheless, Bryant & Haselton [23] found no changes in these parameters across the menstrual cycle. Çelik et al. [42] also failed to find any acoustic changes, but self-reported vocal quality did vary across the cycle, with the lowest quality reported by women in the luteal phase.

There are several methodological aspects to consider to address these inconsistent findings. First, it is noticeable that the sample size is small in some of the studies [24, 41, 42, 44], with only seven or fewer participants in some of them (39, 47), which could have resulted in insufficient statistical power to detect subtle vocal changes. Second, methods used to identify cycle phases vary between studies; researchers have relied on participant’s self-reports of cycle length, and used cycle counting methods [39, 40, 43, 44, 46, 47] or other less precise methods, such as measuring body temperature [45] to identify the cycle phases. In studies where cycle phases were identified with more reliable methods, such as direct assessment of hormone levels or confirmation of the luteinizing hormone (LH) surge in mid-cycle [23, 25], voice was measured in only two cycle phases (follicular and luteal). As Fischer et al. [24] pointed out, while difference in any voice characteristic between the two phases could be suggestive, it is not possible to conclude that it conveys information about women’s fertility status, because the value of this characteristic in the rest of the cycle (e.g. in the menstrual phase) is unknown. Finally, a direct comparison of voice between women with natural cycles and users of hormonal contraception (HC) is lacking. This comparison might be valuable in examining the assumption that vocal changes across the cycle are due to fluctuations in sex hormone levels. Hormonal profiles of women who use monophasic contraceptive pills, where all pills contain the same concentrations of sex hormones, are stable across the cycle [51, 52]. Hence, any difference in voice between HC users and non-users in different stages of menstrual cycle could be attributed to sex hormone fluctuations.

Considering all these methodological aspects, the purpose of this study was to investigate sexually dimorphic vocal features and quality of voice in different phases of menstrual cycle, among naturally cycling women and HC users. It was predicted that the values of sexually dimorphic vocal characteristics, as well as vocal quality, would be higher in the late follicular, compared to the menstrual and luteal phases, in women with natural menstrual cycles. Furthermore, considering the stable hormonal profile of HC users, no systematic changes in their vocal characteristics were expected. Regarding between-group comparisons, it was predicted that naturally cycling women would have higher values of sexually dimorphic traits and voice quality than HC users, especially so during the late follicular phase, which is characterized by increased estrogen levels in non-users.

## Method

### Participants

All participants gave written informed consent to voluntarily participate in this study. The Ethic Committee of Research Involving Humans at the Faculty of Humanities and Social Sciences of University of Zagreb in Croatia approved the study design. Participants were 62 women with regular menstrual cycles (between 24 and 35 days in length) and who had not taken hormonal contraceptives or any other kind of hormonal therapy within the previous three months, and 21 women using monophasic hormonal contraceptives and who had been using these for at least 3 months prior to the start of the study.

All women were students at the University of Zadar, Croatia. They had no diagnosis of any kind of endocrine (e.g. polycystic ovaries, endometriosis) and respiratory problems (e.g. chronic asthma) or speech impairments (e.g. rhotacism). All of them reported being non-smokers, native speakers of the Croatian language, and not being involved in any kind of professional or amateur singing education or training. Of the 62 naturally cycling women, data from ten were omitted due to the LH surge not being detectable, four were omitted because the high fertility session took place more than three days before the LH surge, two due to personal reasons (e.g. moving to another city) and one for reporting respiratory health problems (allergic asthma) during the study. In addition, voice recordings of one participant were discarded due to technical issues during the recording. Of 21 women who were using hormonal contraceptives, one was omitted because she stopped using birth control during the study.

The final sample included in analyses thus consisted of 44 naturally cycling women with an average cycle length of 29.29 days (SD = 2.99), and 20 users of hormonal contraceptives, with 19 of these using combined monophasic pills and one using monophasic patches. It is worth noting that pills and patches regulate menstrual cycle in the same manner; patches release the same daily concentration of synthetic hormones, as does the pill [53]. All users had been using hormonal contraceptives for between 3 and 48 months (M_month_ = 17.75, SD = 14.42) before the study started. All brands of hormonal contraceptives used by women in this study contained greater concentration of synthetic progesterone (gestodene or drospirenone, 3 mg) than synthetic estrogens (ethinylestradiol, from 0.02–0.035 mg).

In addition, it is worth mentioning that the smaller number of contraceptive users than naturally cycling women in this study was due to relatively low prevalence of hormonal birth control usage in Croatia. By collecting information on health profiles of more than 700 female students at the University of Zadar during sample recruitment, data showed that less than 10% were using hormonal contraception, which is in line with the prevalence of this kind of birth control in southeast Europe [54]. Considering that the users of this form of contraception have less variability of menstrual cycle length and stable hormone levels throughout the cycle, a smaller sample size of this group was justified.

Mean age of the participants was 21.64 years (*SD* = 1.79, range 19–25 years), with no significant age difference between the groups (*t* = 0.03, *df* = 62, *p* = .978).

### Identification of menstrual cycle phases

Voice recordings were obtained in the menstrual, late follicular and luteal phases. Natural cycle phases were identified by reverse counting method, with LH surge confirmation using LH test kits. Since self-reported measures based on the women's recall of cycle length are less reliable [55, 56], women with natural menstrual cycles were instructed to track their cycles for at least 3 months before study onset. They were also instructed to send the exact dates of menses onset to the researcher via email each month. Based on this, the researcher calculated the length of the shortest cycle in the past period for every woman and used this information to estimate the date of the next period. The session in late-follicular phase was scheduled 15 days before this date, while the start date of the LH testing was scheduled 17 days before the next period. The LH surge was assessed with a commercially available urine ovulation kit (*Ovugnost*) that was given to participants on their first visit to the laboratory. Participants were given instructions for using LH tests at home, and asked to continue testing until they obtained a positive result. When this occurred, participants brought the positive test to the laboratory for inspection. All voice recordings in the late follicular phase were obtained between 3 days before and 2 days after the LH surge (M = 0.05 days; SD = 1.20). An LH surge precedes ovulation by 24–48 hours, which means that late follicular sessions took place during the fertile window [57, 58].

Voice samples in the luteal phase were obtained after ovulation and before the next menses. On average, luteal sessions took place 7.07 days prior to actual menstrual onset (SD = 1.95).

Collection of voice samples in the menstrual phase was scheduled following self-report of menses onset, and was on the third or fourth cycle day, when sex hormone levels are lowest [57, 58]. The first two days of the cycle were omitted in order to avoid possible symptoms like heavy bleeding or pain, which are more pronounced during the first 48 hours of the cycle [59].

After all three sessions, women reported the date of the onset of the next period, by which researcher confirmed that the voice sample were collected in targeted phases. Voice recording sessions scheduled for users of hormonal contraception corresponded to those scheduled for naturally cycling women. The equivalent session to the menstrual phase was held on the third or fourth cycle day, in late follicular phase 15 days prior the expected onset of next period, which was between the 11^th^ and 16^th^ cycle day (M = 13.24, SD = 1.27) The session in the luteal phase was scheduled between the 20^th^ and 23^rd^ cycle day (M = 21.40, SD = 1.10).

Session order was counterbalanced across participants. Each woman began the sessions in one phase and then proceeded in order through the remaining phases. Among women in the natural cycle group, 9 of them started in the late follicular phase, 14 in the luteal and 21 in the menstrual phase. Among users of hormonal contraceptives, 6 of them started in the late follicular, 5 in the luteal, and 9 in the menstrual phase.

### Procedure

Voice recording sessions took place in a soundproof room. At the beginning of each session, the researcher checked if women showed any symptoms of respiratory problems, upper airway infection, or symptoms of flu or cold. If a woman showed no symptoms, she was instructed to drink one glass of water, after which the recording started. Voices were recorded using a Zoom H4N digital recorder, featuring built-in condenser microphones in an X-Y stereo pattern, and placed at fixed distance of approximately 40 cm from the participant's mouth. A constant recording sound level was used. The recording sampling rate was set for 48 kHz at 24-bit amplitude quantization.

Participants were instructed to produce five monophthong vowels (/a/ as in ‘bar’, /e/ as in ‘let’, /i/ as in ‘bee’, /o/ as in ‘cold’ and /u/ as in ‘you’). Recordings were stored and uncompressed WAV format was used in voice analyses.

## Results

### Acoustical analysis

Voice samples were analysed using *Praat* software (version 5.3.51) [60]. Pitch floor and ceiling were 100 Hz and 500 Hz, in accordance with the programmers' recommendations for analysing female voice [60]; otherwise, default settings were used. All recordings were first inspected aurally and visually for quality, and to ensure no other sounds were present.

Following previous research [23, 30], sexually dimorphic vocal characteristics: fundamental frequency (F0), its variability (F0 SD), minimum (F_0min_), and maximum values (F_0max_) were averaged across the sequence of vowels /a/, /e/, /i/, /o/ and /u/. Characteristics related to vocal quality, perturbation measurements (jitter and shimmer) and HNR were measured on the vowel /a/ phonation. Voice intensity (dB), which corresponds to the loudness of the sound, was also assessed by averaging intensity values across the sequence of vowels.

### Effects of session order on vocal characteristics

Before the main analysis, the potential effect of session order on voice was examined. There were three different session orders in this study. However, an equal number of women with 3 different session orders was not accomplished. For example, there were 19 women with natural cycles who had their first session in the menstrual, then in the late follicular and luteal phases. However, there were only 12 who had the first session in the late follicular phase. In order to examine whether session order had an effect on voice changes in different phases of the menstrual cycle, analyses using repeated general linear models on each of the dependent variables of interest (vocal characteristics) were conducted, with the cycle phase as a within-subject factor, and session order as a between-subject factor. Analysis showed no significant main effects of session order, nor any interaction effects of session order and cycle phase on vocal characteristics (all *p*s > .05).

### Voice changes across the menstrual cycle

Before the analyses, univariate normality was tested for all variables, and Skewness and Kurtosis indices (see Kline’s criteria for normal distribution [61]) pointed to a normal distribution for all variables. In order to investigate vocal changes across the cycle, analyses using repeated general linear models on each of the vocal characteristics were conducted, with cycle phase as a within-subject factor. Analyses were conducted separately for women with natural cycles and HC users.

Descriptive values of vocal characteristics for women with natural cycles and the analysis results are presented in Table 1.

**Table 1:** Changes in vocal characteristics across the menstrual cycle among women with natural cycles.

	Menstrual cycle phase								
	Menstrual		Late follicular		Luteal				
	M	SD	M	SD	M	SD	F	p	pη^2^
F_0_	196.24	19.36	195.16	17.99	196.76	16.82	0.17	0.844	0.004
F_0_ SD	36.54	16.38	30.27	13.71	33.46	13.05	2.60	0.080	0.057
F_0min_	116.10	23.92	131.12	30.62	123.10	25.31	4.94	**0.009**	0.103
F_0max_	316.77	78.85	309.78	61.54	314.41	60.85	0.14	0.872	0.003
Jitter	1.58	0.62	1.51	0.72	1.60	0.67	0.23	0.791	0.005
Shimmer	0.90	0.28	0.86	0.29	0.86	0.22	0.35	0.709	0.008
HNR	12.96	2.29	13.09	2.61	12.75	2.12	0.27	0.762	0.006
Intensity	54.08	2.78	53.73	3.07	53.03	2.66	5.10	**0.008**	0.106

F_0_—fundamental frequency, F_0_ SD—standard deviation of fundamental frequency, F_0min_—minimum values of fundamental frequency, F_0max_—maximum values of fundamental frequency, HNR—harmonics-to-noise ratio, M—mean values, SD—standard deviation, pη2 –partial eta squared, df = 2 for all analyses. Significant effects are in bold.

Analyses showed significant changes in F_0min_ and voice intensity across the menstrual cycle, both with the large effect sizes. Post hoc analysis (LSD tests) revealed that women had higher F_0min_ in the late follicular compared to the menstrual phase (p = .002), while the difference between late follicular and luteal phases did not reach a statistically significant level (p = .097). There was no difference between menstrual and luteal phases (p = .147). Generally, women had higher values of this sexually dimorphic acoustic parameter in the late follicular phase, suggesting more feminine voice during their fertile period.

Furthermore, voice intensity was lowest in the luteal phase. Post-hoc analysis revealed that voice intensity was significantly lower in the luteal compared to menstrual (p = .002) and late follicular phase (p = .040). There was no difference between menstrual and late follicular phases (p = .297). All other acoustic parameters were stable across the cycle.

Next, voice changes across the cycle among HC users were analysed. Descriptive values of vocal characteristics of users of HC, and the results of the analyses, are presented in Table 2. As expected, among HC users, there were no voice changes across the cycle.

**Table 2:** Changes in vocal characteristics across the menstrual cycle among users of hormonal contraceptives.

	Menstrual cycle phase								
	Menstrual		Late follicular		Luteal				
	M	SD	M	SD	M	SD	F	p	pη^2^
F_0_	205.00	24.92	202.59	23.28	204.43	21.19	0.20	0.820	0.010
F_0_ SD	35.72	16.71	35.99	15.86	31.23	15.17	0.62	0.541	0.032
F_0min_	126.33	37.18	115.88	25.57	133.75	35.90	1.43	0.252	0.070
F_0max_	327.97	66.96	322.62	93.77	316.14	78.70	0.14	0.871	0.007
Jitter	1.71	0.77	1.56	0.60	1.40	0.64	1.23	0.302	0.061
Shimmer	0.90	0.24	0.91	0.21	0.84	0.24	0.82	0.448	0.041
HNR	11.60	2.10	11.83	2.48	12.59	1.82	2.51	0.095	0.117
Intensity	53.66	3.28	53.22	2.86	52.93	2.58	0.73	0.491	0.037

F_0_—fundamental frequency, F_0_ SD—standard deviation of fundamental frequency, F_0min_—minimum values of fundamental frequency, F_0max_—maximum values of fundamental frequency, HNR—harmonics-to-noise ratio, M—mean values, SD—standard deviation, pη2 –partial eta squared, df = 2 for all analyses

In subsequent analyses, differences in voice characteristics between the two groups of women were tested. Due to the differences in sample size between the groups and significant Leven’s tests (*p* < .05) that pointed to unequal variances between groups, vocal characteristics of the two groups were directly compared by using corrected F ratios given by Welch test. F ratios were obtained for each vocal characteristic in each menstrual cycle phase. Results are presented in Table 3.

**Table 3:** Differences in vocal characteristics between women with natural cycles and users of hormonal contraceptives.

	Menstrual cycle phase								
	Menstrual			Late follicular			Luteal		
	F	p	pη^2^	F	p	pη^2^	F	P	pη^2^
F_0_	1.94	0.174	0.036	1.60	0.216	0.030	2.04	0.164	0.038
F_0_ SD	0.03	0.856	0.000	1.94	0.173	0.034	0.32	0.574	0.006
F_0min_	1.28	0.269	0.028	4.30	**0.044**	0.057	1.44	0.241	0.029
F_0max_	0.34	0.561	0.005	0.31	0.580	0.007	0.01	0.931	0.000
Jitter	0.44	0.513	0.008	0.09	0.764	0.001	1.31	0.259	0.020
Shimmer	0.00	0.954	0.000	0.59	0.446	0.007	0.14	0.714	0.003
HNR	5.39	**0.025**	0.075	3.43	0.072	0.051	0.09	0.768	0.001
Intensity	0.25	0.619	0.005	0.41	0.524	0.006	0.02	0.892	0.000

F_0_—fundamental frequency, F_0_ SD—standard deviation of fundamental frequency, F_0min_—minimum values of fundamental frequency, F_0max_—maximum values of fundamental frequency, HNR—harmonics-to-noise ratio, M—mean values, SD—standard deviation, pη2 –partial eta squared, df = 1 for all analyses. Significant effects are in bold

Comparison of the two groups of women revealed that the two groups of women differed in their F_0min_ in the late follicular phase. Naturally cycling women produced a higher minimum pitch compared with HC users. Furthermore, women in the natural cycle group had higher values of HNR, but only in the menstrual phase. The difference in the late follicular phase approached the threshold for statistical significance (p = .072), although pη^2^ pointed to a medium effect size. This result indicates that the natural cycle group had higher quality of voice with less noise, which was most pronounced in the menstrual phase.

## Discussion

This study examined voice changes among naturally cycling women and HC users in three phases of the menstrual cycle. There are three key findings. First, results showed some voice changes across the natural menstrual cycle. Second, as expected, there were no changes in vocal characteristics across the cycle in HC users. Third, HC users and non-users differed in minimum pitch and HNR, with all differences pointing to more feminine voice with less noise among women with natural cycles.

Broadly speaking, these results offer support for the assumption that natural fluctuation of sex hormones might be the mechanism underlying voice changes across the menstrual cycle. However, only two vocal parameters changed significantly—F_0min_ and voice intensity. As expected, non-users had lower minimum pitch in the late follicular compared to the menstrual phase, which indicates a stereotypically more feminine voice during the fertile period. However, F_0min_ in the fertile period did not differ significantly from the luteal phase. Therefore, it is not possible to make any strong conclusion about whether this is directly related to fertility status. Nonetheless, it is noteworthy that changes in F_0min_ values in this study correspond to changes in estrogen levels across the cycle. Estrogen levels are lowest in the menstrual phase, and highest in the late follicular phase. Similarly, lowest F_0min_ was found in the menstrual phase, highest F_0min_ in the late follicular phase, with values in the luteal phase being intermediate but more similar to those observed in the late follicular phase. Increased estrogen levels might have had a proliferative effect on laryngeal mucus [22, 39], hence enabling production of higher-pitched tones in the late follicular phase. On the contrary, decrease in estrogen levels might cause decrease in F_0min_ by changing musical secretions and vocal cord muscles tonicity. It is clear, however, that without direct hormone level assessment it is not possible to make any strong claims about this proposed mechanism, especially because the difference in F_0min_ between late follicular and luteal phase was not significant.

Furthermore, women had lowest voice intensity in the luteal phase. Changes in voice intensity cannot be *directly* explained by hormonal influence on the vocal folds because intensity depends neither on vocal fold length nor mucosa with receptors for sex hormones. However, an *indirect* hormonal effect is possible. Voice intensity depends on airstream pressure from the lungs. Pressure depends on activation and arousal, where lower activity decreases the pressure, and higher activity increases it. The latter is usually observed in high emotional states [20]. Since progesterone levels are known to decrease activation levels through regulation of inhibitory GABA neurotransmitters and cortisol levels [62, 63], lower activation in the luteal phase might lead to lower airstream pressure and lower voice intensity. However, in a rare study of voice intensity changes across the menstrual cycle, Tatar et al. [46] found no significant changes. Thus, future investigation of this acoustic feature is warranted.

It is worth reiterating that described effects were observed among naturally cycling women, but were absent among HC users. This implies that voice changes are regulated by sex hormones fluctuations, possibly either through direct influence on vocal cords, or indirectly by regulation of activation level.

Regarding the voice differences between groups, it was expected that women with natural cycles would have more feminine voice and higher vocal quality, especially when groups were compared in the late follicular phase. The results confirmed the initial assumption only with respect to F_0min_, which was higher during the fertile period among naturally cycling women. This implies their greater vocal femininity and ability to produce higher pitched tones in comparison with HC users. Since vocal femininity is related to higher estrogen levels, differences between groups could be explained by increased estrogen levels in the late follicular phase in natural cycles, and the almost 90% decrease of natural estrogen in cycles regulated by HC [52]. However, it is noteworthy that, in the fertile period, groups differed only in F_0min_, while other sexually dimorphic vocal parameters remained the same for both groups.

Groups significantly differed in HNR values in the menstrual phase and marginally significantly in the late follicular phase. Both differences indicate greater vocal quality with less noise among women with natural cycles. It is worth noting that previous studies have found an opposite difference in voice quality between HC users and non-users [39, 47]. Previous researchers reported that pill users had greater vocal quality, assessed by jitter, shimmer [39, 47], and HNR parameters [39], arguing that the relatively stable hormonal profile of HC users leads to greater vocal quality. However, they reported an overall group effect, regardless of the cycle phases, and used smaller sample sizes than in the present study, with only five [47] and seven [39] women in each group.

Furthermore, it is worth noting that hormonal levels were not directly measured in the present study. Therefore, it is not possible to make any strong claims about direct influence of sex hormones on voice changes across the cycle, especially because of great intra- and inter-individual variability in the menstrual cycle length and levels of steroid hormones [64]. The importance of direct assessment of steroid hormones in studying fertility-related changes in highlighted in a recent study by Marcinkowska et al., [65]. By daily measuring of steroid hormones in saliva samples throughout the entire menstrual cycle, authors did not find support for cycle-related changes in preference for male masculine traits. Results of this study suggest that the direct assessment of sex hormones in studying mate choice and changes related to fertility status in necessary.

In summary, significant vocal changes across the menstrual cycle and differences in vocal quality between women with natural cycles and HC users are mostly in line with the initial predictions. However, only few vocal characteristics significantly varied across the cycle. These results, combined with mixed findings in previous studies, suggest that vocal changes across the menstrual cycle are quite subtle. Based on the present findings, it is not possible to make strong inferences about voice as a source of fertility status information, at least not by analysing voice during vowel production. Vowels represent the most common and classical form of vocal stimulus used in acoustic analysis, because this methodology has some important advantages. First, some acoustic parameters can only be measured during vowel phonation (jitter, shimmer, HNR). Second, this methodology enables control for many other factors that could affect voice parameters, such as semantic meaning, intonation, and variability due to different utterances. Thus, it increases experimental control. However, human communication is far more complex than vowel phonation. Hence, it is possible that additional vocal changes might be detected by analysing meaningful sentences recorded in a defined social context. Starting from the main assumption that voice changes in the fertile phase have a function in mating behavior, future studies should look for vocal changes in a mating context at various stages of menstrual cycle. Recently, Karthikeyan and Locke [25], in the first study on cycle-related speech changes in a defined mating context, attempted to detect changes in women’s voice pitch during voice messages recorded towards a masculine and a feminine man in fertile and non-fertile cycle phases. These authors simulated a speed dating context and presented female participants with male voices, manipulated in voice pitch to sound more or less masculine. Surprisingly, they found lower pitch in women’s fertile phase, regardless of the men’s masculinity. There is a possibility that women did not find the men’s voices sufficiently attractive to alter their voice pitch in the predicted manner, or perhaps auditory stimuli were not adequate for a successful simulation of speed dating. Therefore, future studies are needed to address the question of vocal changes in relation to fluctuations of sex hormones in a defined social/mating context, which should perhaps include visual, instead of auditory, stimuli in order to simulate communication between women and potential partners.

## Supporting information

S1 Dataset Vocal characteristics in three phases of menstrual cycle, among women with natural cycle and users of hormonal contraceptives. (XLSX)
